# Novel Roles of the Tim Family in Immune Regulation and Autoimmune Diseases

**DOI:** 10.3389/fimmu.2021.748787

**Published:** 2021-09-17

**Authors:** Yikai Liu, Hongzhi Chen, Zhiying Chen, Junlin Qiu, Haipeng Pang, Zhiguang Zhou

**Affiliations:** National Clinical Research Center for Metabolic Diseases, Key Laboratory of Diabetes Immunology, Ministry of Education, and Department of Metabolism and Endocrinology, The Second Xiangya Hospital of Central South University, Changsha, China

**Keywords:** Tim, autoimmune diseases, multiple sclerosis, rheumatoid arthritis, systemic lupus erythematosus, type 1 diabetes

## Abstract

T cell Ig and mucin domain (Tim) protein family members were identified to be important regulators of the immune response. As their name indicates, Tim proteins were originally considered a T cell-specific markers, and they mainly regulate the responses of T helper cells. However, accumulating evidence indicates that Tims are also expressed on antigen-presenting cells (APCs), such as monocytes, macrophages, dendritic cells (DCs) and B cells, and even plays various roles in natural killer cells (NKs) and mast cells. In recent years, the expression and function of Tims on different cells and the identification of new ligands for the Tim family have suggested that the Tim family plays a crucial role in immune regulation. In addition, the relationship between Tim family gene polymorphisms and susceptibility to several autoimmune diseases has expanded our knowledge of the role of Tim proteins in immune regulation. In this review, we discuss how the Tim family affects immunomodulatory function and the potential role of the Tim family in typical autoimmune diseases, including multiple sclerosis (MS), rheumatoid arthritis (RA), systemic lupus erythematosus (SLE) and type 1 diabetes (T1D). A deeper understanding of the immunoregulatory mechanism of the Tim family might provide new insights into the clinical diagnosis and treatment of autoimmune diseases.

## Introduction

Autoimmune diseases are characterized by abnormal tolerance to self-antigens that cause damage to body tissues (1). The etiology of autoimmune diseases is multifactorial and includes infection, environment and genetics (2–6). Most of these factors have been reported to be associated with immune disorders. Therefore, a better understanding of autoimmune disease pathogenesis is needed to identify better treatments.

T cell Ig and mucin domain (Tim), a transmembrane glycoprotein, has been identified as one of the three human Tim family members (Tim-1, Tim-3, and Tim-4) that play a key role in regulating immunity in conditions such as allergies, asthma, virus infection and transplant tolerance (7–11). In the immune system, Tim-1 has been reported to be preferentially expressed on T helper type 2 (Th2) cells, where it serves as an effective costimulatory molecule for T cell activation (12). Tim-3 was first identified as being expressed on interferon-γ (IFN-γ)-producing Th1 cells. As an inhibitor of inflammatory Th1 cells, Tim-3 interacts with its ligand to cause the death of Th1 cells, thereby reducing IFN-γ production (13). Tim-4 is a natural ligand of Tim-1. Tim-4 is mainly expressed on antigen-presenting cells (APCs), but not on T cells (14), and it participates in autoimmune diseases by regulating the proliferation of T cells (15). However, in recent studies, Tims were shown to be expressed on other immune cell types, such as macrophages, dendritic cells (DCs), natural killer cells (NKs) and B cells (16–19), and may play a critical role in maintaining immune homeostasis. These findings allow us to improve our knowledge of the role of Tims in the immune system. In this review, we focus on the expression and function of Tims on different immune cells, discuss recent studies examining the role of Tims in autoimmune diseases in both animal models and humans, and provide useful insights into the identification of new therapeutic targets.

## The Expression And Function Of Tims On Different Cell Types

**Figure 1:**
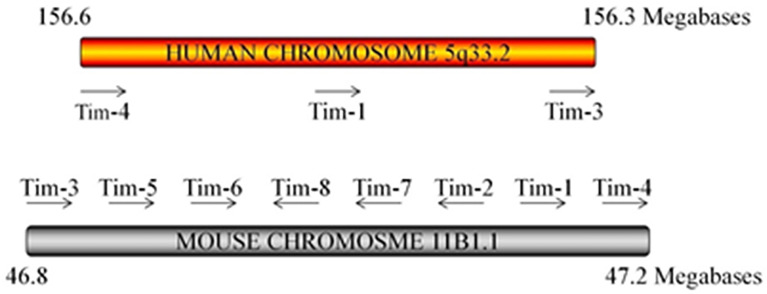
Tim locus. The arrangement of the three Tim genes on human chromosome 5 and the 8 Tim genes on mouse chromosome 11 is shown. Mouse Tim-5–8 are predicted genes. The arrow indicates the direction of mRNA transcription.

**Figure 2:**
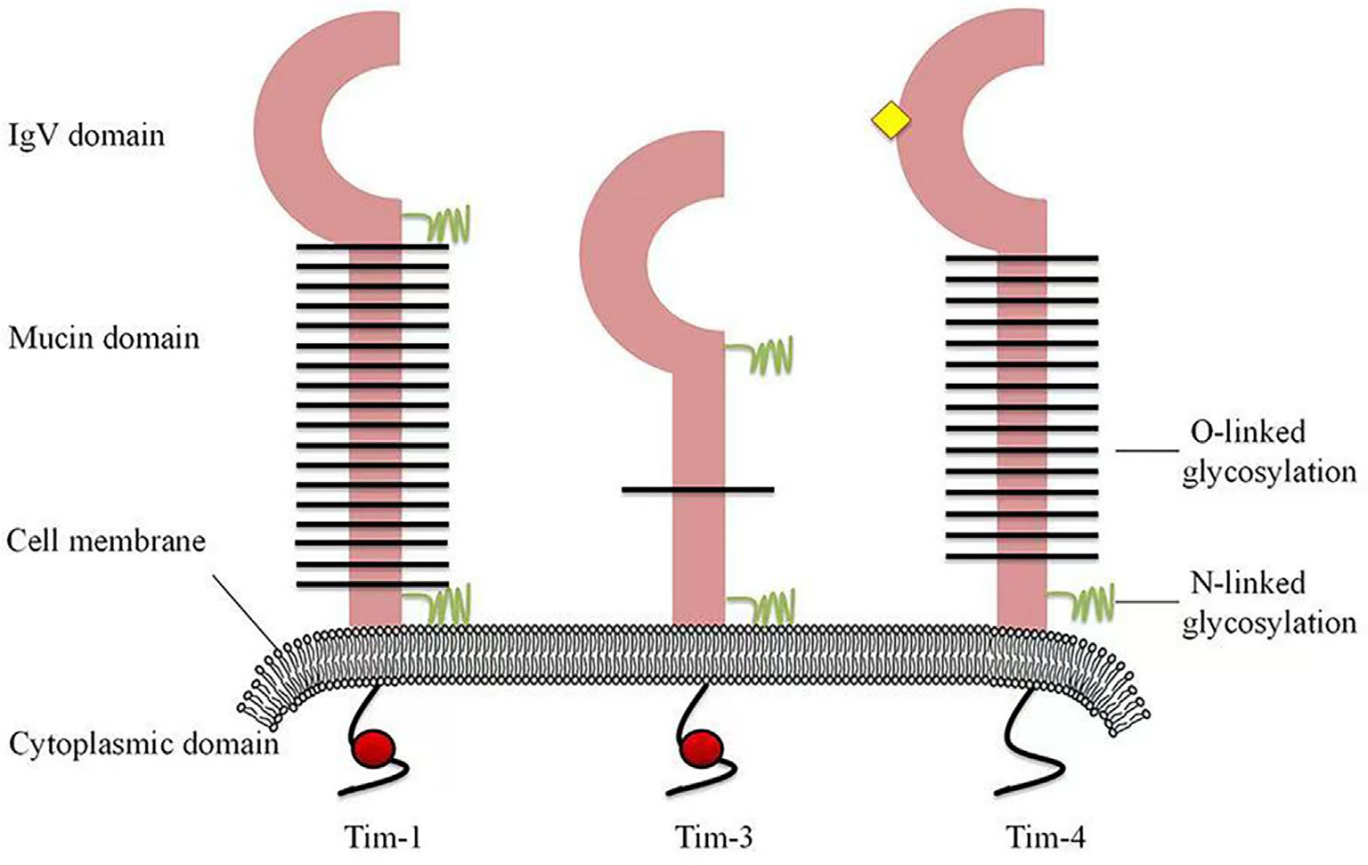
Structures of human Tim family members. Tim proteins contain an IgV domain, a mucin domain, a transmembrane domain and an intracellular domain. Tim-3 has the shortest mucin domain and fewest predicted glycosylated site of the Tims. Tim-4 contains an arginine-glycine-aspartic acid (RGD) motif (yellow diamond), which is present in many ligands that bind to integrins. However, Tim-4 has no tyrosine phosphorylation site (red circle).

Since the discovery of Tims in 2001, major progress has been achieved in terms of elucidating their characteristics and immunological functions (**Table 1**). In mice, the Tim family is composed of eight members (Tim-1 to Tim-8), and the genes that encode them are located on chromosome 11B1.1. In humans, three members (Tim-1, Tim-3, and Tim-4) have been identified, and the genes are located on chromosome 5q33.2 (**Figure 1**) (20). These three human Tim genes are most homologous to mouse Tim-1, Tim-3 and Tim-4, which are associated with allergic diseases. All Tim molecules are type I glycosylated proteins. Tim proteins contain an IgV domain, a mucin domain, a transmembrane domain, and an intracellular domain (**Figure 2**). Tim-1 and Tim-3 contain a tyrosine phosphorylation motif in the intracellular domain. Tim-3 has the shortest mucin domain and fewest predicted glycosylation sites. Tim-4 differs from the other family members, and it contains a short intracellular tail without a tyrosine phosphorylation motif. In addition, Tim-4 possesses an arginine-glycine-aspartic acid (RGD) motif, which is present in many ligands that bind to integrins (20). Therefore, Tim-4 may also act as a decoy receptor. Variations in the structures and motifs of Tim family members indicate that individual Tim proteins may have different roles in signal transduction.

**Table 1:** Known features of the Tim family.

Molecule	Expressing cells	Ligand(s)	Function	Disease	Ref
Tim-1	Activated Th2 cells, Bregs	Tim-4 PS	Costimulation of T cell activation, modulation of Treg function, maintenance and induction of Bregs	Autoimmune diseases, infection, asthma, allergy	(12, 20)
Tim-3	Th1 cells, innate immune cells	Gal-9 HMGB1 Ceacam1 PS	Suppression of the Th1 response, increased activation of signaling pathways leading to T cell activation	Autoimmune diseases, infection, cancer	(13, 21)
Tim-4	APCs	Tim-1 PS	Regulation of T cell proliferation, clearance of apoptotic cells	Autoimmune diseases, chronic metabolic disease, infection, allergy	(15, 22)

### Tim-1

The expression of Tim-1, also known as kidney injury molecule-1 (Kim-1), is highly upregulated on the surface of injured kidney epithelial cells (23). Its expression is increased in urine samples from patients with chronic kidney disease (24). Tim-1 has been used as a biomarker to assess the degree of injury in individuals with acute tubular necrosis. In addition to its role in kidney injury, Tim-1 was also detected in African green monkey kidney cells (6) and later identified in humans as a cellular receptor for hepatitis A virus, called HAVCR-1 (25).

Interestingly, Tim-1 is present on activated but not naive CD4^+^ T cells. Moreover, it is preferentially expressed on activated Th2 cells, but not Th1 cells (12). Tim-1 is a highly effective costimulatory molecule that promotes the formation of T cell receptors (TCRs) through agonistic anti-Tim-1 antibodies, which increase the proliferation of CD4^+^ T cells (12). According to recent studies, Tim-1 has a dual function as a T cell costimulator; it positively or negatively costimulates the T cell response according to the way it engages with T cells during T cell activation (26). Researchers tested a series of monoclonal antibodies (mAbs) against Tim-1 and identified two antibodies targeting Tim-1 that display distinct effects. One agonistic mAb (3B3) increases the production of the proinflammatory cytokines IFN-γ and IL-17, increasing the severity of experimental autoimmune encephalomyelitis (EAE). In contrast, the antagonistic mAb RMT1-10 inhibits IFN-γ and IL-17 production, alleviates the development of autoimmunity (26).

In addition to exerting regulatory effects on Th cells, Tim-1 is also vital for the function and development of regulatory T cells (Tregs). The anti-Tim-1 mAb 3B3 reduces forkhead box protein P3 (Foxp3) expression, prevents effector T cells (Teffs) from differentiating into Tregs and regulates the suppressive ability of Tregs, thereby preventing transplantation tolerance in mice (11). Tim-1 signaling in B cells plays an important role in maintaining the stability of the immune system and inhibiting autoimmune diseases. Recently, researchers generated Tim-1-mutant [Tim-1 (Δmucin)] mice. Notably, the ability of regulatory B cells (Bregs) to produce IL-10 was compromised in these mutant mice (27). B cells with defective Tim-1 or Tim-1 mutations show reduced IL-10 production and increased production of proinflammatory cytokines (28). Based on these studies, Tim-1 expressed in B cells participates in suppressing immune rejection. Other studies have also found that the expression of Tim-3 and Tim-1 on the surface of mouse mast cells promotes the secretion of the inflammatory factors IL-13, IL-6 and IL-4 (29). Thus, Tim-1 plays wide-ranging roles in various cells to regulate the immune system.

### Tim-3

Tim-3 was first reported to be expressed on IFN-γ-producing Th1 cells (21). Binding of Tim-3 to its ligand terminates Th1 immune responses, and Tim-3 expression is regulated by the transcription factor T-bet (30). Human Tim-3 shares 63% amino acid homology with mouse Tim-3. Mouse Tim-3 consists of 281 amino acid residues, while its human homolog consists of 302 amino acid residues (31). The membrane-bound form of Tim-3 includes an N-terminal IgV domain, a mucin domain, a transmembrane domain and a short cytoplasmic tail.

To date, Tim-3 expression has been detected on both innate and adaptive immune cells, such as DCs (32), mast cells (29), macrophages (33), NK cells (34), and CD4^+^ T and CD8^+^ T cells (35). In addition, Tim-3 may be expressed on Th17 cells, although at lower levels than in Th1 cells (36). Subsequently, Tim-3 was also shown to be expressed on Tregs and participate in immune regulation (37). The differential expression of Tim-3 on both innate and adaptive immune cells suggests that Tim-3 exerts different effects on the functions of these cells. In a published study, an anti-Tim-3 Ab was used to block Tim-3 signaling in a mouse model of autoimmune heart disease. The decreased expression of Tim-3 and CD80 on mast cells and macrophages reduces the level of cytotoxic T lymphocyte-associated protein 4 (CTLA-4) on the surface of CD4^+^ T cells, resulting in a decrease in the number of Treg cells and aggravated myocarditis (38). These studies indicate that the Tim-3 signaling pathway affects the adaptive immune system by influencing the innate immune system.

### Tim-4

Tim-4 contains an extracellular IgV domain, a glycosylated mucin domain and an intracellular tail, and it is mainly expressed on APCs, including DCs, NKT cells, B1 cells and macrophages (22).

According to the current knowledge of Tim-4, the interaction between Tim-4 and its ligand plays an important role in the initiation of Th2 polarization. In DCs exposed to cholera toxin (CT) or/and peanut extract (PE), Tim-4 expression is increased and participates in triggering specific Th2 polarization and intestinal allergies (39, 40). Tim-4 is a natural ligand for Tim-1, and emerging evidence suggests that the binding of Tim-1 to Tim-4 is involved in regulating T cell proliferation (41). Interestingly, the dose of Tim-4 is very important for the fate of T cells. High doses of Tim-4 promote T cell proliferation, while low doses exert the opposite effect. The explanation for these effects may be that Tim-4 binds unknown ligands with higher affinity than Tim-1 on T cells, transmitting negative signals. Alternatively, the interaction of Tim-1-Tim-4 may transmit a negative signal at a lower ligand density, similar to the agonist-antagonist phenomenon observed when peptide ligands are changed, and this inhibition is reversed by higher Tim-4 concentrations (41, 42). Another study showed that Tim-4 inhibits the activation of naive rather than activated T cells. Since Tim-1 is not expressed on naive T cells, the inhibitory effect of Tim-4 may depend on unknown ligands other than Tim-1. Thus, Tim-4 has at least two types of ligands: one that promotes T cell activation and another that inhibits T cell activation. These results suggested that Tim-4 might regulate T cell depending on the activation status of T cells, probably by binding different ligands (14). A recent study reported that Tim-4 inhibits the production of nitric oxide (NO) and cytokines in LPS-treated macrophages by inhibiting the nuclear factor kappa B (NF-κB) pathway or janus activating kinase 2 (Jak2)/signal transducer and activator of transcription 1 (STAT1) signaling (43). In summary, Tim-4 is expressed on different cell types and plays various roles in regulating immunity.

## The Ligands Of Tims

Published studies have shown that the soluble Tim-4-Ig fusion protein specifically binds to Tim-1-transfected cells, while the soluble Tim-1-Ig fusion protein also specifically binds to Tim-4-transfected cells. These studies confirm the interaction between Tim-4 and Tim-1. In addition, Tim-4-Ig binds to activated T cells that express Tim-1 at high levels, and this binding is blocked by anti-Tim antibodies. Thus, Tim-4 is indeed the natural ligand of Tim-1 (41). Another study showed that Tim-4 binds to (phosphatidylserine) PS exposed on the surface of apoptotic cells. Hence, PS was identified as another ligand of Tim-4 (44). However, a recent study failed to detect a direct interaction between Tim-1 and Tim-4 (45). Miyanishi et al. showed that Ba/F3 B cells expressing Tim-1 or Tim-4 bind to exosomes through PS. PS is present on the surface of exosomes and is involved in signal transduction between cells (44). This finding indicates that the Tim-1-Tim-4 interaction occurs through the PS bridge. Therefore, the interaction between Tim-1 and Tim-4 is indirect.

Adequate research has confirmed that Galectin-9 (Gal-9) is recognized as a ligand of Tim-3. It binds to the carbohydrate structure of the IgV domain of Tim-3, which contains two N-glycosylation sites. The interaction between Gal-9 and Tim-3 triggers the death of Th1 cells, thereby inhibiting tissue inflammation and inhibiting the progression of EAE (13). Carcinoembryonic antigen cell adhesion molecule 1 (Ceacam1), with a molecular weight of 60 kDa, was recently characterized as another candidate Tim-3 ligand that binds to the Tim-3 IgV domain (46). Ceacam1 is expressed on activated T cells and involved in T cell suppression (47). Tim-3 and Ceacam1 are coexpressed and form a heterodimer. This coexpression is necessary for the inhibitory function of Tim-3. Ceacam1 forms heterodimer interactions in cis or in trans through its N-terminal domains, and both cis and trans interactions between Ceacam1 and Tim-3 affect the immune tolerance of T cells (46). In addition, high-mobility group box 1 (HMGB1) and PS have also been identified as Tim-3 ligands. HMGB1 is mainly related to inhibition of the innate immune response, while PS is related to the clearance of apoptotic cells (48, 49). An understanding of how these ligands coordinate their interactions with Tim-3 and regulate immunity is important.

As discussed above, PS has been identified as a ligand of Tim-4. The crystal structure of Tim-4 showed that the CC’ and FG loops in the IgV domain of Tim-4 create a narrow cavity. A metal ion-dependent ligand binding site (MILIBS) is specifically responsible for the recognition of PS (50). The hydrophilic head of PS penetrates into the MILIBS, its acidic phosphate group is coordinated with metal ions, and the fatty acid tail of PS interacts with the aromatic residues of the FG loop. The hydrophobic residues in the FG loop are essential for PS recognition (51). The single deletion of aromatic residues in the FG loop reduces the binding of the Tim protein to PS in liposomes by approximately 70%, while the double mutation completely eliminates PS binding (50).

In addition, PS is a phospholipid present on dying cells and is a typical “eat me” signal. Tim-4 specifically bind to PS exposed on the surface of apoptotic bodies (AB) *via* the IgV domain, and then mediate engulfment by macrophages (44). Effective clearance of apoptotic bodies maintains normal tissue homeostasis in organisms. The blockade of Tim-4 binding to PS leads to deficient clearance of apoptotic cells and results in systemic autoimmunity.

## Tim Family In Autoimmune Disease

The Tim family plays an important role in regulating immunity. The Tim family has also been reported to exert immunomodulatory effects on many autoimmune diseases. Here, we use multiple sclerosis (MS), rheumatoid arthritis (RA), systemic lupus erythematosus (SLE) and type 1 diabetes (T1D) as examples to summarize the roles of the Tim family in autoimmune diseases (summarized in **Table 2**).

**Table 2:** Studies examining the roles of Tim family members in autoimmune diseases.

Tim	Autoimmune disease	Conclusion	Ref
Tim-1	MS	Tim-1^-/-^ B cell mice developed more severe EAE. Transfer of Tim-1^+^ B cells reduced the severity of EAE in mice.	(28)
	RA	A polymorphism in the Tim-1 gene was related to RA in a Chinese Hui population, and a polymorphism of the Tim-1 promoter region may be related to the susceptibility to RA in Korean populations.	(52, 53)
	SLE	Tim-1 expression in PBMCs was increased in patients with SLE compared with healthy controls and was positively correlated with IL-10 expression.	(54)
	T1D	The numbers of Tim-1^+^ Tregs and Tim-4^+^ Tregs in patients with T1D and NOD mice were significantly reduced.	(55)
Tim-3	MS	Tim-3 expression in PBMCs from patients with MS helped predict the prognosis of the disease. Higher Tim-3 expression was associated with a better prognosis than lower Tim-3 expression.	(56)
	RA	Increased expression of Tim-3 in peripheral blood T cells from patients with RA was negatively correlated with the DAS28 and plasma TNF-α levels.	(57)
	SLE	The expression of Tim-3 and Gal-9 in T cells was increased in patients with SLE compared with healthy controls.	(58)
	T1D	In mice treated with a Gal-9 plasmid, inflammation of the pancreatic islets was reduced, and the number of Th1 cells was significantly reduced.	(59)
Tim-4	MS	Tim-4 has been shown to play a critical role in the T cell-mediated immune response.	(14, 60)
	RA	Increased expression of Tim-3 in peripheral blood T cells from patients with RA was negatively correlated with the DAS28 and plasma TNF-α levels.	(61)
	SLE	The Tim-4 mRNA was expressed at significantly higher levels in PBMCs from patients with SLE than in PBMCs from healthy controls and was positively correlated with Tim-1 mRNA and serum TNF-α levels.	(62)
	T1D	The numbers of Tim-1^+^ Tregs and Tim-4^+^ Tregs in patients with T1D and NOD mice were significantly reduced.	(55)

### The Signaling Pathway of Tims

Tim-1 antibodies were used to identify the signaling pathway by which Tim-1 activates T cells. Overexpression of Tim-1 leads to nuclear factor of activated T-cells (NFAT)/activatorprotein-1(AP-1) transcriptional activation, which depends on Y276 in the cytoplasmic tail of Tim-1 (63). In addition, Tim-1 is recruited to the TCR signaling complex in human T cells through its interaction with CD3. The increase in signaling events related to TCRs include the phosphorylation of Zap70 and IL-2 inducible T cell kinase (ITK). In addition, ITK and phosphoinositide 3-kinase (PI3K) complexes are recruited to the TCR signaling complex (64). After a tyrosine in Tim-1 is phosphorylated in a Lck-dependent manner, the p85 linker subunit of PI3K is directly recruited, leading to PI3K activation (65). Based on these studies, Tim-1-mediated T cell activation may require PI3K activation.

Tim-3 was identified as specifically expressed on the surface of CD4^+^ and CD8^+^ T cells. Studies have found that the tyrosine residues in the cytoplasmic region of Tim-3 are related to T cell signaling (31). When Tim-3 does not bind to its ligand, Tyr256/Tyr263 in the cytoplasmic region of Tim-3 interacts with HLA-B associated transcript 3 (Bat3), and Bat3 recruits the tyrosine kinases Lck to maintains T cell activation (66). However, when Tim-3 binds to the Gal-9, the phosphorylation of Tyr256 and Tyr263 is triggered by ITK (67), then releases Bat3 from Tim-3 and inhibits the T cell signaling by tyrosine kinase Fyn recruitment (68).

Tim-4 has been shown to increase the levels of p-extracellular regulated kinase (ERK) 1/2 and p-Akt in CD3^+^ T cells by cross-linking with Tim-1. Treatment of naive T cells with inhibitory Tim-4-Ig reduces the phosphorylation of linker for activation of T cells (LAT) and ERK 1/2 (14). In addition, Tim-4 inhibits the mitogen-activated protein kinase (MAPK) pathway in T cells.

In summary, Tim proteins participate in the regulation of many signaling pathways, most of which are related to the pathogenesis of autoimmune diseases. Therefore, an understanding of the role of Tims in different autoimmune diseases and their possible signaling pathways and mechanisms will provide new insights to improve immunotherapy. However, the mechanisms by which Tims regulate autoimmune diseases through these signaling pathways are not yet fully understood, and more research is needed to achieve continuous improvements before clinical treatment.

### Tims and MS

Most evidence for the roles of Tims in autoimmune diseases has been derived from studies of mouse EAE models. The findings from these studies enable us to understand the effect of autoimmune pathology, especially regarding the T cell inflammatory response in the central nervous system (CNS) (69). MS is an autoimmune disease characterized by inflammation of the white matter in the CNS. This disease most commonly affects the white matter around the ventricle, optic nerve, spinal cord, brain stem and cerebellum (70). EAE is a mouse model of MS. The pathogenesis of EAE is similar to that of MS, which provides new insights into the pathology of MS.

Xiao showed that a Tim-1 deficiency in B lymphocytes disrupts the balance between regulatory and proinflammatory cytokines in B cells. Mice with Tim-1^-/-^ B cells exhibit an enhanced pathogenic Th1/Th17 response, a decreased number of Foxp3^+^ Tregs and reduced IL-10 expression in CNS-derived T cells, resulting in a worse EAE clinical score (28). In addition, the adoptive transfer of Tim-1+ B cells not only alleviates EAE in wild-type mice but also decreases the severity of EAE in the Tim-1^-/-^ B cell mouse model, showing that Tim-1 is associated with the severity of EAE by regulating the balance between pathogenic T cells and protective Tregs. Tim-4 has been shown to play a critical role in the T cell-mediated immune response. On the one hand, treatment with a Tim-4 blocking antibody *in vivo* reduces the T cell-mediated inflammatory response produced in EAE mice (14). On the other hand, the Tim-4-Fc fusion protein inhibits the activation of naive T cells *in vitro* by inhibiting the activation of the MAPK pathway, inhibits the differentiation of Th17 cells and prevents IL-17 production. Notably, the inhibitory effect of the Tim-4-Fc fusion protein is independent of Tim-1 and requires IgV and mucin domains (60). Based on these studies, Tim-4 has a bimodal regulatory function that depends on the activation status of T cells: an inhibitory effect of Tim-4 on naive T cells and a positive regulatory effect on activated T cells.

Tim-3 is expressed on CD4^+^ Th1 cells that secrete IFN-γ (21). It also ameliorates the symptoms of EAE by inducing the death of pathogenic Th1 cells, and inhibition of Tim-3 aggravates the symptoms of EAE. The expression of both Gal-9 and Tim-3 on Th1 cells *in vitro* induces Th1 cell death and ameliorates EAE (13). During the induction of EAE, the administration of Gal-9 *in vivo* reduces T cell proliferation and IFN-γ production, changes related to reductions in disease morbidity and mortality. In contrast, inhibition of Gal-9 *in vivo* with an siRNA exacerbates the development of EAE. Dysregulation of Tim-3 expression in MS has been reported in clinical studies. Koguchi et al. studied CD4^+^ T cell clones isolated from the CSF of patients with MS. Compared with CD4^+^ T cells from healthy controls, CD4^+^ T cells from patients with MS express lower levels of Tim-3 and produce more IFN-γ (71). Tim-3 signaling also induces the death of specific CD8^+^ T cells, and the use of Tim-3-blocking antibodies exacerbates CD8^+^ T cell-mediated EAE (72). Yang et al. examined Tim-3 function on CD4^+^ T cells isolated from the circulatory system of healthy controls and patients with MS. Blocking Tim-3 during T cell stimulation significantly promotes the secretion of IFN-γ in healthy controls. Tim-3 inhibition has no effect on treated patients, suggesting that patients with MS have defects in Tim-3-mediated immunoregulation (73). According to recent studies, the expression levels of Tim-3 on peripheral blood mononuclear cells (PBMCs) from patients with MS help predict the prognosis of the disease. Lower expression levels of Tim-3 on PBMCs are associated with an increased possibility of progression to secondary progressive multiple sclerosis (SPMS), while higher Tim-3 expression levels on PBMCs are associated with a benign prognosis 10 years later (56).

### Tims and RA

RA is a systemic inflammatory autoimmune disease characterized by joint pain and swelling. In severe cases, it can lead to joint deformities and loss of function (74). RA affects 0.5–1% of the adult population and is more common in women (75). Although the pathogenesis of RA remains elusive, multiple factors are widely accepted to be involved. Genetic, environmental and hormonal factors may all contribute to the pathogenesis of RA (76, 77).

Recently, polymorphisms in Tim genes were reported to be potential risk factors for RA. Xu et al. reported that Tim-1 (-1637A>G, -232A>G), Tim-3 (-1541C>T, +4259G>T) and Tim-4 (SNP rs7700944) gene polymorphisms are related to RA susceptibility in the Chinese Hui population (52, 78, 79). Similar results were also reported for other national populations (53, 80). Tim-4 is involved in the immunoregulation of collagen-induced arthritis (CIA), and it exhibits dual functions, depending on the phase of CIA. During the induction phase, treatment with anti-Tim-4 monoclonal antibodies exacerbates the development of CIA in mice. In contrast, during the effector phase, treatment with anti-Tim-4 monoclonal antibodies reduces proinflammatory cytokine levels in the ankle joint, significantly inhibiting the progression of CIA (61).

Tim-3 may be a potential new marker for assessing the severity of RA. The expression levels of Tim-3 on PBMCs from patients with RA have been reported. Liu et al. showed increased expression of Tim-3 on peripheral blood CD4^+^ T cells, CD8^+^ T cells, NKT cells and monocytes from patients with RA. The percentage of Tim-3^+^ cells is negatively correlated with the 28-joint disease activity score (DAS28) and plasma tumor necrosis factor alpha (TNF-α) levels (57). In another study, Tim-3 expression on CD4^+^ and CD8^+^ T cells was shown to be negatively correlated with the progression of RA (81). In addition, the number of Tim-3^+^ Foxp3^+^ Tregs is decreased in patients with RA and is negatively correlated with RA disease activity (82). Based on accumulating evidence, the Tim-3-Gal-9 pathway may play an essential role in the induction and development of RA, and it may be a clinical target for the treatment and alleviation of RA. In an animal model, treatment with Gal-9 was shown to induce naive T cells to differentiate into Tregs, not only reducing the production of proinflammatory cytokines in mouse joints but also decreasing the number of Tim-3^+^ CD4^+^ T cells in the peripheral blood (83). In a clinical study, significantly higher Gal-9 expression in several T cell subsets and plasma was observed in patients with RA than in healthy controls. After 12 weeks of treatment with a calcineurin inhibitor, Gal-9 expression levels in individuals with a good therapeutic response were significantly lower than in those with a poor therapeutic response (84). Tim-3 is considered a useful biomarker for determining disease activity and progression. However, the current knowledge on Tim-3-targeted therapy for RA is still limited, and more studies in humans are required to provide further evidence (85).

### Tims and SLE

SLE is an autoimmune disease with diverse clinical manifestations involving multiple organs. Its etiology is unclear, and it is related to various factors, including genetic, immune, and hormonal factors (86–89).

Lupus nephritis is the main risk factor for the overall morbidity and mortality of SLE and is related to the dysregulation of Th1 and Th2 responses (90). Tim-1 has an important role in regulating the Th1/Th2 response (91). Studies of mouse models of nephritis have suggested that when an inhibitory anti-Tim-1 antibody (RMT1-10) is administered to mice with nephritis, Foxp3^+^ T cells accumulate in the mice, and the expression of the IL-10 mRNA increases. RMT1-10 treatment reduces the urinary excretion and renal expression of Tim-1, reflecting an alleviation of interstitial injury (92). Previous studies have suggested that patients with SLE show increased expression of Tim-1 in PBMCs compared with healthy people, and Tim-1 expression is positively correlated with IL-10 expression. Moreover, this study also found significantly increased levels of the Tim-1 mRNA in patients with active SLE (SLE disease activity index (SLEDAI)>6), which indicates that Tim-1 mRNA expression in PBMCs is related to the disease activity of patients with SLE (54). Interestingly, in another study, researchers found that the mRNA expression levels of Tim-4 and Tim-1 were positively correlated in patients with SLE, but this correlation was not obvious in healthy controls. However, the authors failed to detect a significant difference in the Tim-1 mRNA expression levels between patients with SLE and healthy controls. This difference may be due to the distinctive SLEDAI of the subjects participating in each study (62). In summary, unlike other Tim molecules, research on the role of Tim-1 in SLE is still limited, and more research is needed to explore this protein in the future.

Th1 and Th17 immune dysregulation is one of the causes of SLE (93). Tim-3 was initially identified on activated Th1 and Th17 cells and induced T cell death after binding to its ligand, Gal-9 (94). Jiao et al. investigated the expression of Tim-3 and Gal-9 in patients with SLE and healthy controls. The expression of Tim-3 and Gal-9 on various T cells (including CD4^+^ T cells, CD8^+^ T cells, and CD56^+^ T cells) was significantly higher in patients with SLE than in healthy controls (58). Another study indicated that the plasma level of soluble Tim-3 (sTim-3) was increased in patients with SLE and positively correlated with anti-dsDNA antibodies, SLEDAI score, erythrocyte sedimentation rate (ESR), and urine albumin levels (95). All these studies illustrated that Tim-3 is potentially useful as an effective biomarker for evaluating indicators of SLE disease activity.

Similar to RA, insufficient clearance of AB is also a cause of SLE. If ABs are not engulfed by macrophages or DCs, the antigens and harmful substances from ABs will trigger an immune response, thereby promoting the progression of SLE (96, 97). The elimination of ABs is a key mechanism for maintaining normal tissue homeostasis in multicellular organisms. Tim-4 binds to PS and exposes it on the surface of ABs, presenting a signal to macrophages to trigger engulfment. Tim-4 mediates the clearance of ABs by macrophages. The mechanism of apoptotic cell phagocytosis is as follows: Ba/F3 transformants expressing the Tim-4 complex and integrin α(v)β (3) bind to and phagocytose apoptotic cells in the presence of milk fat globular epidermal growth factor VIII (MFG-E8) (98). A recent study showed that mice lacking Tim-4 or MFG-E8 rarely develop antibodies (99). In contrast, mice lacking both Tim-4 and MFG-E8 produce high levels of anti-dsDNA antibodies, indicating that Tim-4 and MFG-E8 mediates the clearance of apoptotic bodies, and involved in pathogenesis of SLE (15). In a human study, Zhao et al. observed significantly higher Tim-4 mRNA levels in the PBMCs from patients with SLE, especially those in the active phase of the disease, than those in healthy controls. Moreover, the level of the Tim-4 mRNA in PBMCs from patients with SLE positively correlated with the expression of the Tim-1 mRNA and serum TNF-α levels (62). Overexpressed Tim-4 may bind to Tim-1 and promote a Th2-mediated immune response, especially in patients with SLE. TNF-α is mainly secreted by activated macrophages and may induce an increase in Tim-4 expression, thereby promoting the proliferation of Th2 cells by binding to Tim-1. These findings imply that Tim-4 exerts a regulatory function in the pathogenesis of SLE.

### Tims and T1D

T1D is a chronic autoimmune disease that is mainly caused by the destruction of islet β cells mediated by T lymphocytes (100). Due to the continuous destruction of insulin-producing islet β cells, insulin deficiency and hyperglycemia occur. Patients with an uncontrolled disease may suffer from ketoacidosis, which can be life-threatening (101). Noninsulin-based treatment strategies, such as delaying β cell failure, stem cell treatment, and islet transplantation, would be optimal to ameliorate T1D in patients and prevent its complications (102–104).

Research by Shimokawa confirmed that CD8^+^ Tregs are essential for preventing autoimmune diabetes. Notably, compared with healthy individuals, patients with T1D have fewer CD8^+^ Tregs (105). Tim-1 and Tim-4 are considered essential for the activation and differentiation of T lymphocytes. Guo et al. evaluated the expression of Tim-1 and Tim-4 in Tregs and found that the numbers of Tim-1^+^ Tregs and Tim-4^+^ Tregs were significantly decreased in both patients with T1D and no obesity diabetes (NOD) mice (55).

The Tim-3 pathway represents an important mechanism for downregulating Th1-mediated autoimmune diseases and promoting the development of immune tolerance. A previous study showed that treating recipient mice with a Tim-3-specific monoclonal antibody accelerated the occurrence of autoimmune diabetes in an adoptive transfer NOD model. In addition, similar results were obtained when researchers treated recipient mice with the Tim-3-Ig fusion protein, which disrupts the integrity of the inhibitory interaction between Tim-3 and its ligand on T cells (106). Blockade of the Tim-3 pathway accelerates diabetes in NOD mice. This effect may be mediated by inhibiting the immunosuppressive function of Tregs. Furthermore, Gal-9 was identified as a ligand for Tim-3 and shown to suppress the Th1 immune response in the development of T1D (107). Compared with control mice, NOD mice overexpressing the Gal-9 were significantly protected from T1D and showed less inflammation of pancreatic islets (59). Many studies have shown that the Tim-3 pathway is involved in Th1-mediated disease, and blocking the signaling by Tim-3 and its ligand Gal-9 may aggravate autoimmune diseases, including T1D (108).

## Conclusions

Considerable progress has been achieved in understanding the expression and function of the Tim family in autoimmune diseases. Tim proteins are intimately involved in immunoregulation and participate in many diseases, such as allergies, infections, and cancers, by influencing the immune system. The data on the Tim family also provide us with insights into the design of selective targeted therapeutics. Clearly, the expression of Tim molecules is not limited to T cells, indicating that they perform different functions in a variety of cells to modulate immune responses. Emerging evidence suggests that Tim-1 has a potential role in the maintenance and regulation of Bregs. Tim-3 negatively regulates the response of Th1 cells and inhibits the production of inflammatory factors. Similarly, Tim-4 seems to play a positive role in clearing apoptotic cells and might participate in systemic autoimmune diseases. Therefore, future research on the Tim family is expected to provide new strategies for autoimmune treatment.

## Author Contributions 

YL searched the references, wrote the first draft of the paper and revised the text. HC, ZC, JQ, and HP critically revised the text and provided substantial scientific contributions. ZZ proposed the project and revised the manuscript. All authors contributed to the article and approved the submitted version.

## Funding

This study was supported by the National Natural Science Foundation of China (grant numbers 8181001262 and 81970746) and the Natural Science Foundation of China (grant number 82000748).

## Conflict of Interest

The authors declare that the research was conducted in the absence of any commercial or financial relationships that could be construed as a potential conflict of interest.

## Publisher’s Note

All claims expressed in this article are solely those of the authors and do not necessarily represent those of their affiliated organizations, or those of the publisher, the editors and the reviewers. Any product that may be evaluated in this article, or claim that may be made by its manufacturer, is not guaranteed or endorsed by the publisher.
